# How does chronic pain impact the lives of dogs: an investigation of factors that are associated with pain using the Animal Welfare Assessment Grid

**DOI:** 10.3389/fvets.2024.1374858

**Published:** 2024-04-04

**Authors:** Rachel Malkani, Sharmini Paramasivam, Sarah Wolfensohn

**Affiliations:** School of Veterinary Medicine, University of Surrey, Guildford, United Kingdom

**Keywords:** dog, welfare assessment, quality of life, chronic pain, veterinary medicine

## Abstract

**Introduction:**

Chronic pain can profoundly affect the wellbeing of dogs and our understanding is limited regarding the multidimensional impact it has on dog quality of life. This study aimed to assess the factors that are significant and predictive of chronic pain in dogs using the Animal Welfare Assessment Grid (AWAG) to further understand what factors influence their welfare.

**Methods:**

Seventy six AWAG assessments were undertaken across 46 dogs that clinicians diagnosed as having musculoskeletal conditions that caused chronic pain. Wilcoxon-rank sum tests were used to assess the difference in scores between dogs with chronic pain and a cohort of healthy dogs (*n* = 143).

**Results:**

All physical factors besides body condition, and all psychological, environmental, and procedural factors were significantly different between healthy dogs and dogs with chronic pain, evidencing how chronic pain impacts all domains of a dog’s life. Spearman Rank Correlation Coefficient (RS) revealed several significant strong positive correlations such as the association between the severity of clinical symptoms with poorer mobility and the frequency at which the dog experienced fearful stimuli. Logistic regression showed that fears and anxieties frequency, the dog’s reaction to stressors, engagement with enrichment, and social interactions were significant predictors of chronic pain in dogs.

**Discussion:**

This highlights that typical signs of musculoskeletal disorders such as gait changes, stiffness, lameness might manifest after behavioral changes such as increased fearfulness, prolonged recovery from a stressful event, a reduced interested in social interactions, toys or play. Owners only seeking veterinary attention when the presence of physical signs of disease are evident may result in a delayed veterinary attention resulting in reduced welfare. Regular veterinary assessments combined with use of the AWAG can proactively identify these behavioral indicators and result in prompt treatment and improved quality of life.

## Introduction

Pain is defined as an unpleasant sensory and emotional experience associated with actual or potential tissue damage; or an aversive sensory and emotional experience typically caused by, or resembling that caused by, actual or potential tissue injury (1).

Pain may be classified in a range of ways according to its duration (acute, chronic, or intermittent) and severity (mild, moderate, severe, excruciating) (2). Chronic pain is described as pain that has persisted for longer than 3–6 months and may have several components. The inciting cause may or may not be present (3). Reorganization of cortical neurotransmitters occurs after injury and this can induce central sensitization, hyperalgesia, and allodynia (4, 5). Changes in the brain as a result of chronic pain can impact cognitive and emotional health, resulting in poor welfare states (6, 7).

Chronic pain does not seem to serve any useful purpose for an animal besides having a protective function, and may be very difficult to recognize behaviorally (2). Chronic pain is notoriously difficult to assess and quantify; the pain that a patient with osteoarthritis might exhibit may bear no resemblance to the radiographic features of the disease (3, 8). However, it is important to prioritize objective assessments aimed at evaluating the quality of life impact of chronic pain, as opposed to solely quantifying the severity of the primary condition.

A well-recognized indication of both acute and chronic pain in dogs is behavior change (9, 10). Reported signs include a general reduction in activity levels, change in appetite, reduced sleep quality and change of sleeping position, altered posture and gait changes that may include stiffness or lameness. These changes are mapped out on the physical parameter of the Animal Welfare Assessment Grid (AWAG) (11). Psychological and environmental factors also included in the AWAG that are reported in the literature to be indicative of pain include aggression, personality changes, reduced sociability and play, and reluctance or refusal to perform normal behaviors (2, 3, 10, 12, 13). Many pain scoring tools include these examples of behavior change coupled with facial expressions to score the severity of pain (14–16).

There have been many scales developed to quantify the severity of acute pain. However, there is no gold standard as the experience is unique to the individual and because of its multidimensional nature. This presents a challenge in assessing the affective component of pain as ideally, a comprehensive evaluation needs to consider physiological, psychological, endocrine, immune, and behavioral measures (17), and this can be further compounded by the assessor’s bias. Multidimensional scales that have been developed for use in dogs include the Glasgow Composite Measure Pain Scale (CMPS) (18, 19), the University of Melbourne Pain Scale (20, 21), and the Colorado State University Canine Acute Pain Scale (22).

In canine chronic pain, studies have highlighted that the owner is the preferred proxy because behavioral changes may be so subtle and gradual in onset that they are apparent only to someone very familiar with the individual dog and these subtle behavioral changes may not be obvious to veterinary professionals in a clinical setting where they may be masked by fear, excitement, or anxiety associated with the unfamiliar environment (10). Therefore, caregiver reports regarding behavioral change are likely to be highly beneficial when assessing chronic pain in a dog. However, owners are reported to focus on movement-based behavior changes in their dogs and have difficulty associating behavioral changes with pain in the older dogs (23), while other studies have shown that when using structured chronic pain assessment tools, owner’s may not be able to identify behavioral changes associated with pain in their dogs (24). Additionally, there is also bias in veterinary assessments of pain. 90% of veterinarians and the public believe that larger breed dogs have less sensitivity to pain, despite there being no physiological basis for this (25). Moreover, female veterinarians are reported to give higher pain scores compared to males (26). Therefore, conducting assessments based on both owners and veterinary input, may provide the most accurate data using chronic pain assessment tools.

As pain can inhibit normal behaviors, not only can the emotional experience of physical pain affect quality of life, but dogs may become frustrated if they cannot perform behaviors as normal. This may be as a result of restrictions imposed by the owner, or the physical inability of the dog to perform these. Frustration occurs when an animal’s expectations and desires are not met and is considered a negative emotional state and potential welfare concern (27).

Dogs that have reduced mobility due to pain and increasing age may result in a decline in owner positive attitude toward their dog and a reduction in the amount of time the owner spends together with their dog (28), which can be frustrating and damaging to the welfare of the dog. In younger dogs with decreased mobility, frustration may be experienced with higher intensity to due increased energy and desire to play, run, and interact socially with other dogs and people.

Monitoring chronic pain using diagnostic tools alone can be problematic. Using physiological parameters as a stand-alone measure risks treatments being directed at controlling and improving that parameter rather than examining what is impacting the animal’s quality of life related to the condition and how to improve it (29).

Pain can be an important consideration in many medical conditions and often has an impact on diagnostic and treatment decision making. Pain is the primary concern in dogs with osteoarthritis and other disorders that cause lameness. Conditions affecting the elbow joint are a common cause of lameness in both young and older dogs; the canine elbow can be affected by several different diseases, including elbow dysplasia, humeral intracondylar fissures, congenital luxations, soft-tissue problems, and septic arthritis (30). Each of these diseases usually results in lameness, joint pain, and reduced elbow movement, that adversely affect welfare.

An exploration of welfare impact among common disorders of dogs in the UK identified osteoarthritis to have the highest severity score and second highest welfare impact, with dental disease having the highest welfare impact (31). A large group of UK veterinary surgeons were surveyed about their concerns regarding chronic pain in dogs. Osteoarthritis was perceived as the most common cause of chronic pain in the dog and vertebral and spinal cord conditions were also perceived as a relatively common cause of chronic pain (14). As osteoarthritis is often multifaceted with several disease processes and genetic factors playing a role in the development of the condition, it is important to understand the effects on welfare and how these can be improved.

In addition to current methods of pain evaluation, the AWAG and similar tools may complement and enhance these to provide a holistic approach to chronic pain assessment. This study aims to use the AWAG to understand the effects of chronic pain on canine welfare. The AWAG is a valid and reliable tool scores a range of factors across the four parameters of physical and psychological health, the environment, and procedural and management events (Table 1). Once the user has scored all factors ranging from one the best welfare possible to ten the worst welfare possible using mutually exclusive written descriptors, the AWAG calculates a cumulative welfare assessment score (CWAS) and a mean score for each parameter. The CWAS is the total area of the polygon which is generated by plotting the mean of each parameter across four axes on a radar chart.

**Table 1:** Factor scores and their written descriptors.

Physical			
Mobility	Body condition	Clinical assessment	Eating and drinking
1. The dog has very good mobility with no lameness or stiffness and is normally active or has normal energy	1. Ribs easily palpable without pressure, with minimal fat covering, waist easily noted and evident abdominal tuck	1. Clinically healthy, no injury or sign of disease	1. Eating and drinking as normal
2. Very good mobility with occasional mild stiffness and is normally active	2. Ribs fairly easy to palpate without pressure with thin fat covering and evident abdominal tuck from above	2. Mild transient subclinical symptoms or injury but has no evident behavior change or impact on welfare	2. Food and/or water consumption is minimally reduced
3. Good mobility with short bouts of stiffness	3. Slight fat covering, slight pressure needed to palpate ribs, waist observable from above	3. Mild transient clinical symptoms or injury with mild transient behavior change and impact on welfare	3. Mild to moderate reduced food/water (>20%)
4. Good mobility with generalized stiffness	4. Slight covering of fat, slight waist observable from above, can palpate ribs with pressure needed	4. Mild clinical symptoms or injury with mild behavior change and impact on welfare	4. Moderately reduced food/water (>30%)
5. Moderate mobility, stiffness but frequently active	5. Moderate covering of fat, waist discerned from above but not prominent, can palpate ribs with pressure	5. Moderate transient clinical symptoms or injury with some behavior change and impact on welfare	5. Moderately reduced food/water (>50%)
6. Moderate mobility, stiffness and less active	6. Excess covering of fat, no discernible waistline and difficulty palpating ribs	6. Moderate clinical symptoms or injury with moderate behavior change and impact on welfare	6. Severely reduced food/water (>80%)
7. Poor mobility, stiffness and less active	7. (Overweight) heavy fat present and slight abdominal distension, difficult to palpate ribs or (underweight) ribs and shoulder visible with little fat	7. Moderate/severe disease or injury with moderate behavior change and impact on welfare	7. Anorexic, has minimal loss of skin turgor
8. Very poor mobility, stiffness and minimally active	8. (Overweight) heavy fat present with abdominal distension, cannot palpate ribs or (underweight) ribs, lumbar vertebrae and pelvic bones somewhat visible with little detectable fat	8. Moderate/severe disease or injury with severe behavior change and impact on welfare	8. Anorexic, has moderate loss of skin turgor, somewhat dry mucous membranes
9. Very poor mobility, stiffness and not at all active	9. (Overweight) very heavy fat present with obvious abdominal distension, cannot palpate ribs or (underweight) ribs, lumbar vertebrae and pelvic bones easily visible with very little fat	9. Severe disease and clinical symptoms or injury with severe of behavior change and impact on welfare	9. Anorexic, has considerable loss of skin turgor, dry mucous membranes OR severe hunger/thirst
10. Non-ambulatory and cannot move without assistance or support	10. Massive fat deposits over neck thorax, spine, limbs and base of tail with obvious abdominal distention, cannot palpate ribs or ribs, lumbar vertebrae, pelvic bones and all bony prominences evident from a distance. No discernible body fat and obvious loss of muscle mass	10. Extreme disease and clinical symptoms or injury with extreme behavior change and impact on welfare	10. Anorexic, has major loss of skin turgor, extremely dry mucous membranes OR severe and constant hunger/thirst

Psychological			
Aggression toward caregiver	Aggression toward unfamiliar people	Fears and anxieties frequency	Reaction to stressors
1. None	1. None	1. Rarely encounters stressors	1. Displays minimal signs of fear and anxiety when encounters potential stressors
2. Occasionally growls, is predictable and trigger avoided	2. Occasionally growls, is predictable and trigger avoided	2. Encounters stressors a couple of times a year	2. Shows signs of fear to stressors and returns to normal <30 s
3. Occasionally growls, is predictable but trigger not always avoided	3. Occasionally growls, is predictable but trigger not always avoided	3. Encounters stressors multiple times a year	3. Shows signs of fear to stressors and returns to normal in minutes
4. Occasionally growls, is predictable but trigger rarely avoided	4. Occasionally growls, is predictable but trigger rarely avoided	4. Encounters stressors monthly	4. Shows signs of fear to stressors and some minor and returns to normal after 10 min
5. Occasionally snaps or bites, is predictable and trigger avoided	5. Occasionally snaps or bites, is predictable and trigger avoided	5. Encounters stressors weekly	5. Shows signs of fear to stressors and returns to normal after 30 min
6. Occasionally snaps or bites, is predictable but trigger not always avoided	6. Occasionally snaps or bites, is predictable but trigger not always avoided	6. Encounters stressors several times weekly	6. Shows signs of fear to stressors and takes up to an hour to return to normal
7. Occasionally snaps or bites, is predictable but trigger rarely avoided	7. Occasionally snaps or bites, is predictable but trigger rarely avoided	7. Encounters stressors daily	7. Shows signs of fear to stressors and takes several hours to return to normal
8. Bites, is somewhat predictable and trigger largely avoided	8. Bites, is somewhat predictable and trigger largely avoided	8. Encounters stressors over 50% of the day	8. Shows signs of fear to stressors and takes most of the day to return to normal
9. Bites, is somewhat predictable and trigger not avoided	9. Bites, is somewhat predictable and trigger not avoided	9. Encounters stressors over 75% of the day	9. Shows signs of fear to stressors and takes several days to return to normal
10. Severe bites that are unpredictable	10. Severe bites that are unpredictable	10. Encounters constant stressors	10. Shows signs of fear to stressors and is always anxious

Environment		
Choice, control, and predictability	Enrichment	Social
1. Has good control over their environment and can make a range of choices, has highly predictable environment	1. Engaged in multiple forms of enrichment for over 2 h daily	1. Has high-quality social interactions daily
2. Has good control over their environment and can make a range of choices, mostly has predictable environment	2. Engaged in multiple forms of enrichment for 1–2 h daily	2. Has high-quality social interactions most days
3. Has some control over environment, can make some choices, has mostly predictable environment	3. Engaged in multiple forms of enrichment for up to 1 h daily	3. Has good-quality social interactions daily
4. Has some control over environment, can make some choices, has some predictability	4. Engaged in enrichment for up to 30 min daily	4. Has good-quality interactions most days
5. Has little control over environment, can make some choices, has little predictability	5. Engaged with enrichment for less than 15 min daily	5. Has good-quality interactions weekly
6. Spends several hours in an unpredictable environment, can make some choices	6. Somewhat engaged with enrichment several times weekly	6. Has moderate-quality interactions weekly
7. Spends half of the day unpredictable environment, can make few choices	7. Somewhat engaged with enrichment weekly	7. The dog is socially isolated most days and has moderate-quality interactions in between
8. Spends most of their time in unpredictable environment, can make few choices	8. Poorly engaged with enrichment monthly	8. The dog is socially isolated most days and has poor social interactions in between
9. Spends the majority of time in unpredictable environment, can make very few choices	9. Rarely engages with any forms of enrichment	9. The dog is socially isolated for 50% of each day and has poor social interactions the rest of the time
10. Spends almost all of their time in highly unpredictable environment, cannot make any choices	10. Has no enrichment or does not engage with enrichment	10. The dog is constantly socially isolated

Procedural			
Behavior during assessment	Change in daily routine	Handling during assessment	Procedure pain
1. Is calm and actively seeks interaction from assessor/s	1. Procedure/disruption to day <15 min	1. Displays minimal signs of stress when handled, is calm and tolerates being handled well	1. No procedure required
2. Is mostly relaxed and shows mild signs of stress to few triggering events	2. Procedure/disruption to day <30 min	2. Minimal movement when handled, sometimes licks lips, yawns or shows other appeasement behavior	2. Minor procedure with no expected pain
3. Is somewhat relaxed and shows mild signs of stress to some triggering events	3. Procedure/disruption to day 30 min–1 h	3. Minimal movement when handled, licks lips, yawns, or shows appeasement behavior frequently	3. Minor procedure longer duration with no expected pain
4. Is not relaxed and shows moderate signs of stress to few triggering events	4. Procedure/disruption to day 1–2 h	4. Some slow movement when handled, turns head away from handler, slow panting, displays more than two signs of stress such as ears back and tail down	4. Minor procedure with short mild pain
5. Shows moderate signs of stress to some triggering events	5. Procedure/disruption to day 3–4 h	5. Moderate movement when handled, fast panting, displays more than two signs of stress such as ears back, tail tucked and furrowed brow	5. Moderate procedure with short duration of transient pain
6. Shows moderate signs of stress to all triggering events	6. Procedure/disruption to day >4 h	6. Some attempt to escape, fast movements, tense body and tense closed mouth	6. Moderate procedure, longer in duration with transient pain
7. Shows major signs of stress to few triggering events	7. Procedure/disruption to day >6 h	7. Moderate attempts to escape, fast movements or frozen and staring, tense and trembling	7. Moderate/severe procedure, with pain lasting >12 h
8. Shows major signs of stress to some triggering events	8. Procedure/disruption to day >8 h	8. Strong attempts to escape when handled or frozen, lifts lips and shows teeth	8. Severe procedure with pain lasting >24 h
9. Shows major signs of stress to all triggering events	9. Procedure/disruption to day >12 h	9. Will violently attempt to escape when handled or frozen, growls and barks	9. Severe procedure with pain or complications lasting >48 h
10. Cannot cope being in the environment, is extremely shut-down or aggressive and shows major signs of stress	10. Procedure/disruption to day >24 h	10. Cannot be handled, growls and attempts to bite when approached	10. Extensive procedure resulting in severe long-term pain or complications

This study will examine the scores between dogs suffering from chronic pain and those deemed physically and emotionally healthy by clinicians. It also aims to explore correlations among the factors to understand how they interconnect, and to determine which factors of welfare might be predictive of chronic pain in dogs. In doing so, this study seeks to offer a comprehensive understanding of the multifaceted impact of chronic pain on dogs, enhancing strategies for effective management and improving overall dog quality of life.

## Methods

The recruitment process for participants in the study involved engaging veterinary surgeons, veterinary nurses, behaviorists, and animal welfare professionals. Individuals were recruited through veterinary networks across the UK. The recruitment strategy encompassed various approaches, including distributing study information posters to partner practices at the University of Surrey and in the Veterinary Times journal. Extensive dissemination of project details were carried out within the researcher’s professional networks. To reach a wider audience, recruitment posters were shared on social networking platforms such as Facebook, Twitter, and LinkedIn from 15/01/2021 to 13/12/2022. This allowed interested individuals to access the recruitment link and share it within their own networks to leverage further dissemination of the AWAG study.

Veterinary surgeons were provided with instructional videos on using the AWAG (accessible via http://awag.org.uk/portal-help). One which provides a systematic walkthrough of how to register and assess a dog, the other on where to see the results and how to interpret these. This provides a consistent and standardized approach among users.

The AWAG^1^ was used to assess the welfare of dogs that were deemed by the assessing clinician to have a musculoskeletal condition that caused chronic pain. Assessments were undertaken from 23 June 2021 to 31 July 2023. Forty six dogs were assessed, and 76 assessments were undertaken across these dogs. Inclusion criteria included any age and breed of dog at any stage of treatment for a disorder that causes chronic pain. The ages of the dogs ranged from 2 years-old to 17 years-old. Chronic pain could be categorized under the heading “musculoskeletal” as arthritis, osteoarthritis, cruciate disease, hip dysplasia, patellar luxation or other; under the other option, a free text box was available to write additional information about the condition. Throughout this study, the term chronic pain refers to any one of these conditions.

The data used for healthy dogs (*n* = 143) were from dogs used in a previous study (11). These dogs were assessed by veterinarians and behavior clinicians to be medically and emotionally healthy.

To determine if there was a significant difference between factor scores of healthy dogs and dogs with a condition that causes chronic pain, Wilcoxon rank-sum test (Mann–Whitney U test) were performed for each factor using R Statistical Software (v4.0.1) (32).

A correlation matrix was performed to examine the relationships between each factor using the cor() function, and the resulting matrix was converted to long format using the melt() function from the reshape2 package (33).

To examine what factors were predictive of chronic pain, a logistic regression model was fitted using the glm() function (34). The model coefficients, *p*-values, and significance levels were extracted using the coef() and format.pval() functions. As several factors were either strongly correlated or were shown to be part of a poorer fitting model using Likelihood Ratio Tests (LRT) and AIC evaluation, eight factors were removed from the model to avoid multicollinearity and to improve model fit. The final logistic regression model formula included:

### Logistic regression model formula

logit(*p*) = *β*0 + *β*1·Aggression toward caregiver+*β*2·Aggression toward unfamiliar people+*β*3·Behavior during assessment+*β*4·Enrichment+*β*5·Fears and anxieties frequency + *β*6·Reaction to stressors+*β*7·Social interactions.

## Results

### Healthy vs. chronic pain

**Figure 1:**
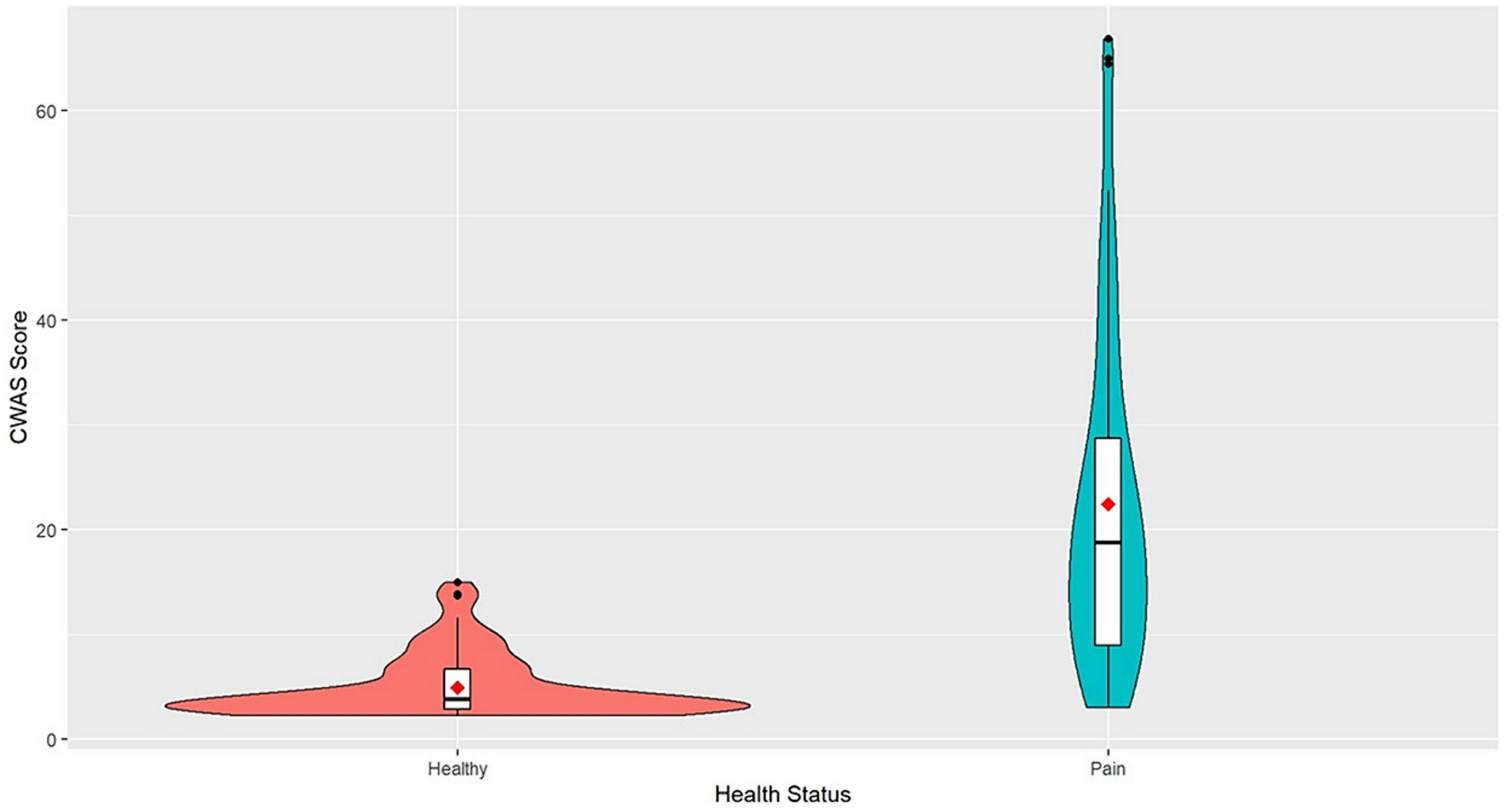
Violin plot of CWAS of healthy dogs and dogs with chronic pain. The violin plot represents the range and concentration of the scores with the widest part showing the highest frequency of scores. The center of each violin plot shows a boxplot with the median line and first and third quartile data on either side. The mean scores are represented as the red point.

The mean CWAS for dogs with conditions that cause chronic pain was 22.47 and ranged from 3 to 66.87. Healthy dogs scored on average 4.94 and ranged from 2.25 to 15 (Figure 1).

**Figure 2:**
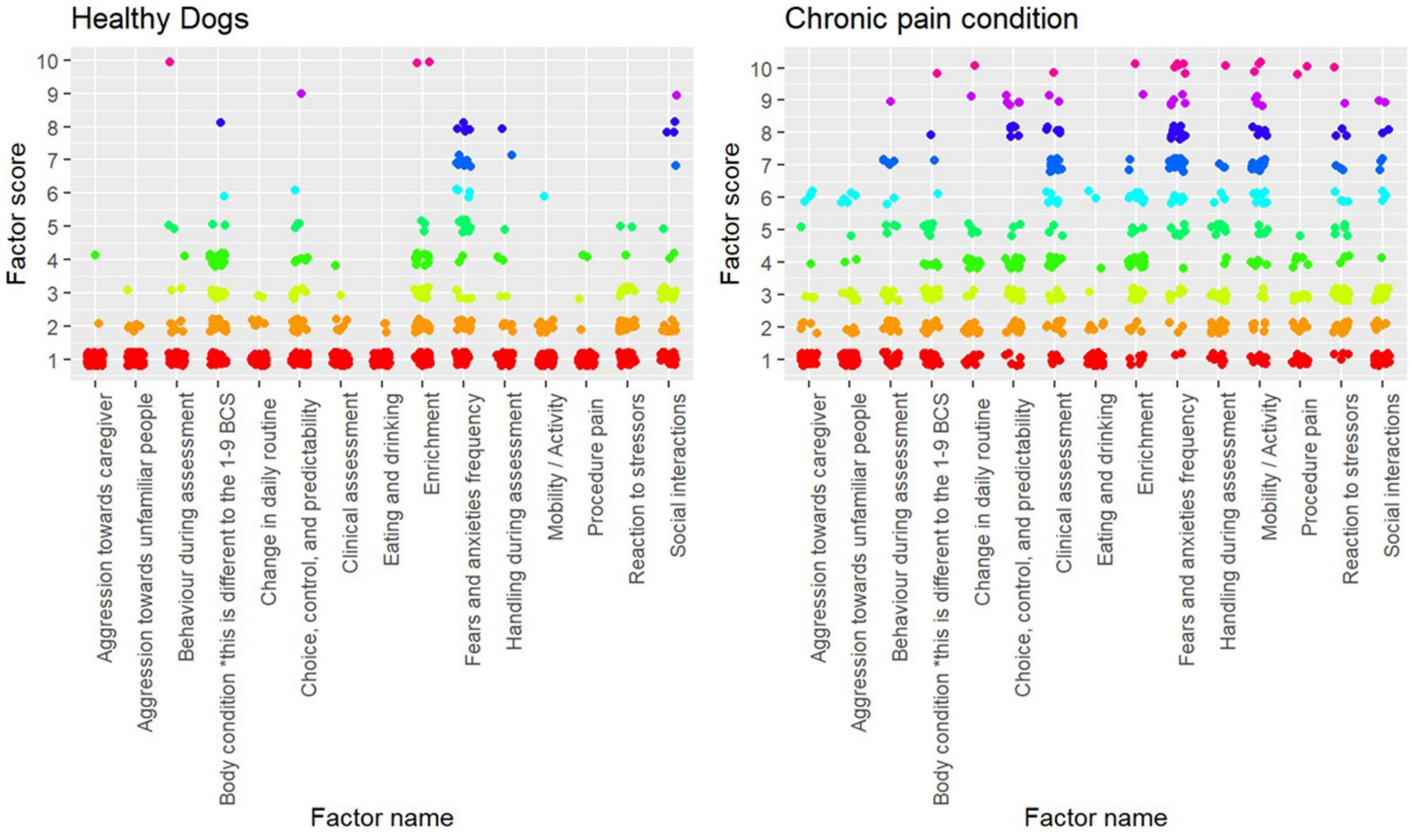
Plot of individual factor scores for each assessment (healthy, *n* = 143; chronic pain, *n* = 76).

Wilcoxon (rank-sum) tests showed that every factor besides body condition significantly different between dogs evaluated to be healthy and dogs with conditions that cause chronic pain (Table 2). The variation between scores can be seen in Figure 2.

**Table 2:** Results of Wilcoxon (rank-sum) tests between healthy dogs and dogs with chronic pain.

Factor	*W* value	*p* value
Aggression toward caregiver	2,079	<0.001
Aggression toward unfamiliar people	2,030	<0.001
Behavior during assessment	866	<0.001
Body condition score	2,363	0.4485
Change in daily routine	330	<0.001
Choice, control, and predictability	1265.5	<0.001
Clinical assessment	337.5	<0.001
Eating and drinking	2,417	<0.001
Enrichment	1,383	<0.001
Fears and anxieties frequency	936.5	<0.001
Handing during assessment	772.5	<0.001
Mobility/activity	450	<0.001
Procedure pain	688.5	<0.001
Reaction to stressors	890	<0.001
Social interactions	2,150	<0.01

### Factor correlation analysis

**Figure 3:**
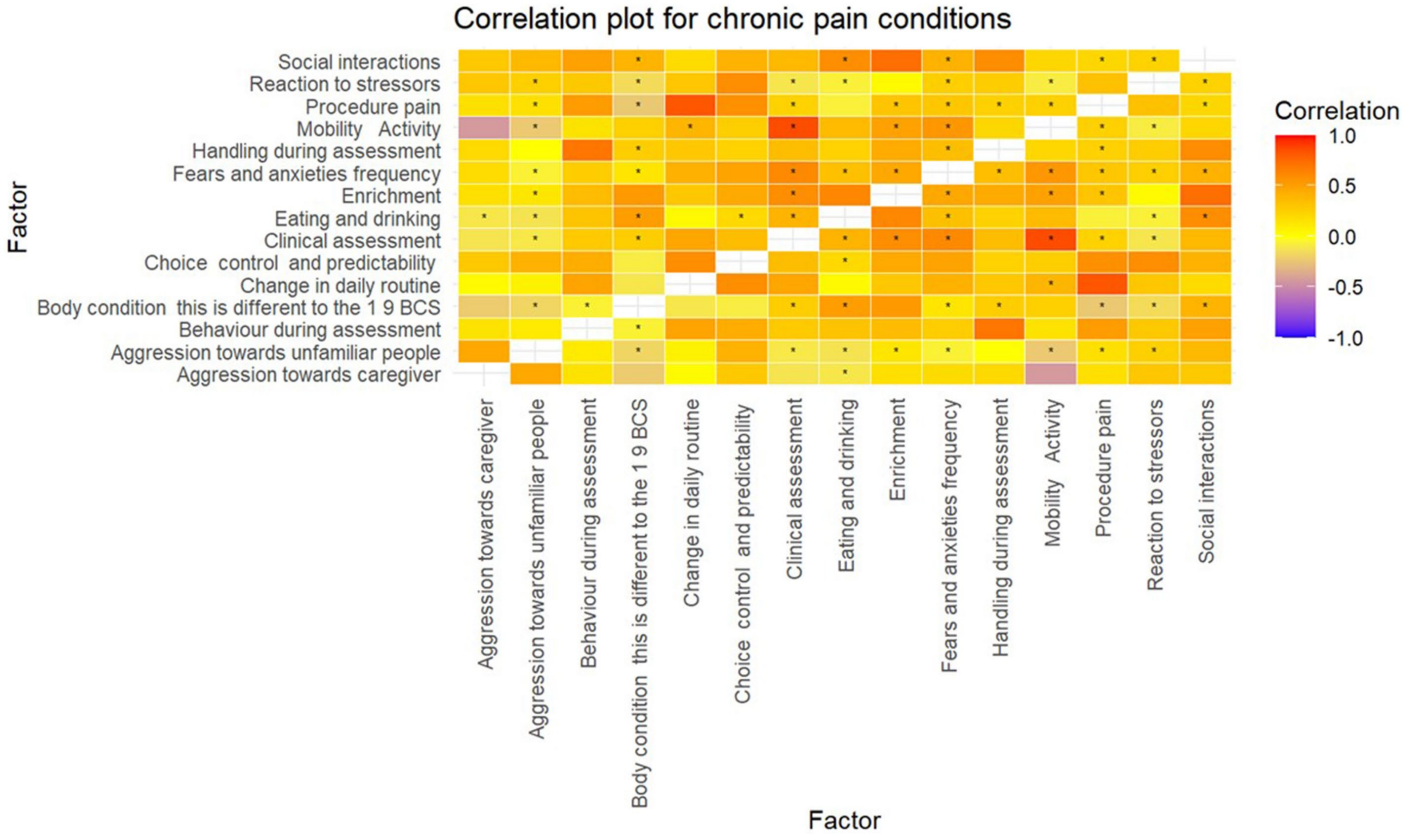
Correlation matrix for conditions that cause chronic pain. *p-value < 0.05.

Several strong (> ± 0.7) positive and negative correlations were found between several factors (Table 3) of which six were statistically significant (Figure 3).

**Table 3:** Table of correlation coefficients (r) from Spearman Rank correlation analysis.

Factor	Aggression toward caregiver	Aggression toward unfamiliar people	Behavior during assessment	Body condition	Change in daily routine	Choice, control, and predictability	Clinical assessment	Eating and drinking	Enrichment	Fears and anxieties frequency	Handling during assessment	Procedure pain	Mobility/activity	Reaction to stressors	Social interactions
Social interactions	0.164	0.014	0.289	0.643	−0.396	−0.279	0.182	0.6	0.614	0.104	0.532	−0.293	−0.046	−0.3	1
Reaction to stressors	0.621	0.654	0.443	−0.789	0.45	0.732	−0.568	−0.736	−0.725	−0.189	−0.089	0.664	−0.518	1	−0.3
Procedure pain	0.036	0.207	0.632	−0.461	0.893	0.832	0.046	−0.332	−0.318	0.314	0.261	1	0.114	0.664	−0.293
Mobility/activity	−0.725	−0.696	−0.132	0.489	0.343	−0.025	0.914	0.607	0.632	0.839	0.118	0.114	1	−0.518	−0.046
Handling during assessment	−0.125	−0.221	0.639	0.443	0.225	0.021	0.354	0.414	0.354	0.3	1	0.261	0.118	−0.089	0.532
Fears and anxieties frequency	−0.346	−0.389	0.143	0.293	0.511	0.214	0.879	0.429	0.514	1	0.3	0.314	0.839	−0.189	0.104
Enrichment	−0.486	−0.493	0.018	0.882	−0.218	−0.4	0.682	0.957	1	0.514	0.354	−0.318	0.632	−0.725	0.614
Eating and drinking	−0.586	−0.625	0.039	0.929	−0.239	−0.45	0.661	1	0.957	0.429	0.414	−0.332	0.607	−0.736	0.6
Clinical assessment	−0.611	−0.643	0.025	0.564	0.296	−0.114	1	0.661	0.682	0.879	0.354	0.046	0.914	−0.568	0.182
Choice, control, and predictability	0.364	0.518	0.336	−0.554	0.696	1	−0.114	−0.45	−0.4	0.214	0.021	0.832	−0.025	0.732	−0.279
Change in daily routine	−0.207	−0.014	0.532	−0.396	1	0.696	0.296	−0.239	−0.218	0.511	0.225	0.893	0.343	0.45	−0.396
Body condition	−0.514	−0.614	−0.082	1	−0.396	−0.554	0.564	0.929	0.882	0.293	0.443	−0.461	0.489	−0.789	0.643
Behavior during assessment	0.075	0.168	1	−0.082	0.532	0.336	0.025	0.039	0.018	0.143	0.639	0.632	−0.132	0.443	0.289
Aggression toward unfamiliar people	0.889	1	0.168	−0.614	−0.014	0.518	−0.643	−0.625	−0.493	−0.389	−0.221	0.207	−0.696	0.654	0.014
Aggression toward caregiver	1	0.889	0.075	−0.514	−0.207	0.364	−0.611	−0.586	−0.486	−0.346	−0.125	0.036	−0.725	0.621	0.164

A significant positive correlation (0.93) was found between the dog’s body condition and eating and drinking, i.e., as the dog’s body condition worsened, so did eating and drinking. Clinical assessment was significantly positively correlated mobility and activity (0.91), and also fears and anxiety frequency (0.88) i.e., the severity of clinical symptoms increased with poorer mobility and the frequency at which the dog experienced fearful stimuli. Additionally, fears and anxiety frequency was also positively correlated with mobility and activity (0.84).

A significant negative correlation (−0.79) was found between the dog’s body condition and reaction to stressors, i.e., as body condition improved, the dog took longer to recover from a stressor. Similarly, eating and drinking and the dog’s reaction to stressors were significantly negatively correlated (−0.74) i.e., as eating and drinking improved, the longer the dog took to normalize from a stressful experience.

### Logistic regression of factors predictive of chronic pain

The logistic regression model showed that fears and anxieties frequency (ß = 0.47, SE = 0.2, *z* = 2.35, *p* = 0.01), reaction to stressors (ß = 1.65, SE = 0.58, z = 2.87, *p* = <0.01), engagement with enrichment (ß = 1.64, SE = 0.48, *z* = 3.43, *p* = <0.001), and social interactions (ß = −1.43, SE = 0.53, *z* = −2.71, *p* = <0.01) were significant predictors of chronic pain in dogs.

## Discussion

This study investigated the CWAS in dogs with conditions that result in chronic pain, explored the factors that are associated with these, and examined which factors may be predictive of conditions that cause long-term pain. Compared to healthy dogs, dogs with chronic pain were scored much higher across all factors and had an average cumulative welfare score of 22.47 indicating poorer overall welfare. Healthy dogs generally had scores clustered around the mean value, whereas dogs with chronic pain showed a much wider variation. The highest CWAS was 66.87 in dogs with chronic pain, this is 51.87 points higher than the highest CWAS for healthy dogs. The scores not only demonstrate the profound impact pain can have on quality on life but show how varied welfare state can be in dogs that suffer from conditions that cause chronic pain. Therefore, the AWAG shows valuable utility in illustrating the variability in quality of life in dogs with chronic pain.

When examining each factor individually, all factors besides body condition were significantly different in dogs with chronic pain compared to healthy dogs. The analysis showing that body condition scores were not statistically different was an interesting finding given that other studies have shown that quality of life is poorer in overweight and obese dogs (35, 36). However, body condition alone is not an indicator of pain other variables contribute to quality of life. Dogs with conditions such as osteoarthritis may have reduced muscle mass or have low energy and stamina, despite maintaining reasonable body condition. Moreover, the poorer AWAG scores include both overweight and underweight dogs reflecting suboptimal body condition whereas the aforementioned studies only examine overweight dogs.

Regarding the other physical factors, it is unsurprising that clinical assessment and mobility and activity were significantly poorer in dogs with chronic pain. Lameness, altered gait, stiffness, and exercise intolerance are widely cited as predictors of chronic pain in the literature (37–39), and these are common motivators to prompt veterinary attention (40). Eating and drinking was found to be poorer in dogs with chronic pain compared to healthy dogs. Although not commonly cited in the literature (41), reduced appetite may be associated with musculoskeletal conditions such as osteoarthritis; but is more commonly linked to other disorders such as cancer and gastrointestinal disease (42, 43) and fear-based behavior problems (44, 45). An alternative explanation may be that owners of dogs with musculoskeletal conditions may attempt to control their dog’s weight through food reduction. These findings may demonstrate that the AWAG can capture vital information about the dog’s quality of life regardless of the condition without having to use more nuanced, disease-specific tools.

Aggression being more prevalent in dogs with chronic pain is consistent with the existing body of literature. Pain and discomfort are reported to lower the threshold for aggressive responses and may also act as a defensive or protective function (12, 46). It is suggested that there are different patterns of pain expression dependent on if the dog has displayed aggression prior to the onset on pain (47). Dogs who previously exhibited no signs of aggression prior to pain are likely to display aggressive behavior more frequently during physical interactions; this should be of importance to clinicians to communicate to dog caregivers that any subtle signs of stress such as yawning, lip-licking, groaning may be indicative of pain during contact. Prior to the behavior escalating into overt aggression, thorough pain investigations and analgesic trials should be undertaken to effectively rule out pain as a differential.

In one study, owners were questioned about their dog’s posture, activity, mobility and behavior and categorized as having chronic pain or not. Dogs presumed to have chronic pain were reported to display protective aggression toward certain body parts and show aggressive behavior toward strangers significantly more often than dogs without signs of chronic pain (23). These findings align with our results where aggression is displayed at higher frequency to both caregivers and strangers where there is evidence of chronic pain.

The dog’s response to stressors and also the frequency at which they encounter these were found to be significantly poorer in dogs with conditions that are associated with musculoskeletal pain. Our results suggest that when faced with a stressor, dogs with chronic pain display signs of fear and anxiety and take longer to recover. Chronic pain is shown to enhance the intensity of behavior through prolonged activation of the hypothalamic–pituitary–adrenal (HPA) axis and the sympatho-adrenal-medullary (SAM) axis resulting in evaluated heart rate, respiratory rate, blood pressure, and blood glucose, cortisol, and catecholamines concentrations (48, 49) If a dog is already is a state of physiological arousal prior to encountering a threat, the stress response will be more intense, thus taking longer to normalize. Additionally, the stress response associated with pain can lead to a reduction in serotonin activity (50), and a reduction in serotonin can be linked to aggressive behavior in dogs (51) Furthermore, as serotonin modulates emotional responses, this may be influencing dogs encountering fears and anxieties at a greater frequency. Thus, under the procedural parameter, it is unsurprising that dogs also displayed more signs of emotional distress during AWAG assessments and when being handled.

All factors in the environmental parameter were also found to be worse in dogs with chronic pain compared to healthy dogs. Dogs with pain had less choice, control, and predictability in their lives and also poorer quality social interactions, which may be a result of caregivers limiting their environment and play opportunities for fear of exacerbating pain. Conversely, dogs with chronic pain may attempt to avoid activities or environments that they associate with discomfort in order to prevent pain, but cannot, resulting in frustration and a loss of control and predictability.

Research in humans demonstrates that a sense of control is important for reducing anxiety and depression (52, 53), and this may provide empowerment and a reduction of helplessness. Similarly, when dogs consistently encounter situations that they have no control over such as chronic pain, it is likely to have a negative impact on emotional state. Furthermore, having little or no predictability in the environment may reduce feelings of security, leading to or increasing anxiety-like states.

Dogs in pain may also withdraw themselves from social interactions with people or other dogs (10, 40) and the AWAG scores demonstrate that dogs with chronic pain have poorer quality social interactions and are more socially isolated compared to healthy dogs, who have higher quality direct engagement with people and/or dogs (dependent on the individual’s needs) that involves training or play. Dogs may avoid direct interaction with their caregivers or other dogs as any physical touch may be painful, particularly play where chasing and exaggerated movements are common (54, 55). Social play is reported to decrease in dogs with chronic pain and may use aggression to end interactions with other dogs (56). Dogs may have learned through classical conditioning that physical engagement results in pain, creating negative associations with dogs and people, leading them to become guarded and withdrawn. This may explain why enrichment and social interactions are not highly correlated; dogs with chronic pain may engage more with object play rather than social play as it allows them greater control the situation and remove themselves when necessary. Additionally, dogs with chronic pain are reported to have more sleep disturbances and spend less time resting (57, 58), which may negatively impact emotional state and sociality, culminating in poor welfare. Alternatively, owners may restrict dogs from engaging in play or other social activities to pain, leading to social isolation.

Dogs with chronic pain engaged with enrichment opportunities less often than healthy dogs. Enrichment in this study is defined as anything in the dog’s environment that enhances their welfare state such as exercise, games, training, toys, or feeding devices. Similar to social interactions, dogs may avoid engagement as a protective function, or owners may limit access to any resources the facilitate movement for fear of exacerbating pain.

In the dogs with conditions that cause chronic pain, several expected correlations were observed. Given the causal relationship between body condition and eating and drinking, and clinical assessment and mobility, it is unsurprising that these are highly correlated. Of particular interest were the correlations between fears and anxiety with mobility and activity and clinical assessment. This demonstrates that as clinical assessment and mobility worsens, dogs encounter fears and anxieties at an increasing frequency. There is growing evidence to suggest that certain fears and anxieties such as fearfulness of loud noises are associated with musculoskeletal pain (56). Pain can result in changes in cognition, and it may alter how the dog perceives their environment. They may become more sensitive to sounds and smells, particularly if they find them aversive. Dogs with poorer mobility may feel a heightened sense of vulnerability and anxiety, due to a reduced ability to escape.

An interesting finding to note was the negative correlation between reaction to stressors and eating and drinking and body condition. This suggests that as body condition and eating and drinking improves, the longer a dog takes to recover from a stressor that indices a negative emotional response. However, it is important to note that the direction of the improvement in body condition was not specified (e.g., underweight to normal or from overweight to normal). As we found that eating and drinking was poorer in dogs with chronic pain, overweight dogs might experience an improvement in body condition through reduced appetite, while their emotional state remains negatively affected by the pain, leading to a heightened stress response and longer recovery. Furthermore, in underweight dogs with chronic pain, other comorbid diseases such as Cushing’s disease (hyperadrenocorticism) may cause polyphagia, resulting in improved body condition alongside chronic stress.

The most compelling results of our study pertain to the factors that are reported to be predictors of chronic pain. The dog’s response to stressors, the frequency at which they experience fear and anxiety inducing stimuli, the dog’s engagement with enrichment, and their social interactions are all shown to be significant predictors of conditions that cause chronic pain. These are of particular importance to veterinary surgeons as these are shown to be evident before any clinical signs appear and any change in these should be regarded as an indicator of a musculoskeletal disorder. Chronic pain originating from bones, nerves, muscles, etc. can be incredibly difficult to diagnose (14, 59). Moreover, the dog may still be highly performing activities they are highly motivated to do such as eat, play and run, despite having underlying pain. Therefore, it is important for clinicians not to assume that pain is not a factor where there are no obvious indicators of chronic pain.

Initial indicators of pain may be that the dog responds to stimuli that previously would not elicit a reaction. They may show subtle signs of stress such as yawning, lip licking, hypervigilance, or momentarily freezing. When encountering situations that would normally induce signs of fear, anxiety, or frustration, the dog may take slightly longer to normalize. Caregivers may notice a reduced interest in engagement with members of the household or other dogs, which if unnoticed, may escalate to repulsion behavior from the dog to avoid interactions. However, it is important to consider that individual differences and personality (60) may influence a dog’s behavioral response to pain. Although this may be more applicable to acute pain rather than chronic. Dogs may also spend less time engaging with enrichment that involves exercise, games, and interactions with toys. The dog may still be motivated to engage with their environment; however, prolonged activity may result in feelings of pain or discomfort, leading to an overall reduction in interaction.

### Limitations

The primary limitations of the study are that all the disorders categorized as musculoskeletal that can cause chronic pain are combined in the analysis together due to insufficient sample sizes for other conditions that can cause pain such as skin disease and cancer. Therefore, we could not assess if specific conditions impacted quality of life differently. Additionally, as assessments were undertaken as various stages of treatment journeys, we cannot examine pre and post treatment across dogs, only at the individual level. As data collection progresses, additional analyses can be undertaken to evaluate the welfare impact of each disease. Additionally, the inclusion of data from dogs with cancer or neurological pain will provide further insights into the influence of pain quality of life. Another limitation is that the scores used in the analysis are from various timepoints ranging from initial presentation to post treatment; therefore, it is unknown what the effects of analgesia or other treatments have on the collective scores. Further research examining the scores of dogs at initial presentation will contribute to a better understanding of how chronic pain impacts dogs prior to any intervention. In addition, investigating human-dog interactions and their influence in pain management will be of value to understand how this dynamic can improve the welfare of dogs with chronic pain.

## Conclusion

In conclusion, this study provides a comprehensive examination of the impact of chronic pain on the welfare of dogs with musculoskeletal conditions. The use of the Animal Welfare Assessment Grid proves to be a valuable tool in capturing the variability in quality of life among dogs with chronic pain and highlights the importance of considering both clinician assessment alongside caregiver reports. The study reveals that chronic pain has a profound and multifaceted effect on various aspects of a dog’s life and interacts with physical health, psychological health, the dog’s environment, and any procedures and management events. The recovery time from a stressor, the frequency at which a dog encounters fear and anxiety-inducing stimuli and the quality of social interactions are shown to predictors of chronic pain. Highlighting the importance of the consideration of pain as a differential, especially in cases where typical clinical signs indicative of chronic pain may not be readily apparent.

## Data availability statement

The raw data supporting the conclusions of this article will be made available by the authors, without undue reservation.

## Ethics statement

The animal studies were approved by NASPA who are a sub-committee of the Animal Welfare and Ethical Review Body (AWERB) University of Surrey. The studies were conducted in accordance with the local legislation and institutional requirements. Written informed consent was obtained from the owners for the participation of their animals in this study.

## Author contributions

RM: Conceptualization, Data curation, Formal analysis, Investigation, Methodology, Project administration, Software, Visualization, Writing – original draft, Writing – review & editing. SP: Supervision, Writing – review & editing. SW: Supervision, Writing – review & editing.

## Footnotes

^1^ http://dog.awag.org.uk
