# Corrigendum: How does chronic pain impact the lives of dogs: an investigation of factors that are associated with pain using the Animal Welfare Assessment Grid

**DOI:** 10.3389/fvets.2024.1414314

**Published:** 2024-05-13

**Authors:** Rachel Malkani, Sharmini Paramasivam, Sarah Wolfensohn

**Affiliations:** School of Veterinary Medicine, University of Surrey, Guildford, United Kingdom

**Keywords:** dog, welfare assessment, quality of life, chronic pain, veterinary medicine

In the published article, there was an error. In the abstract, chronic pain is referred to as behavioural disorders.

A correction has been made to the abstract, *[Introduction and methods***]**. The sentences previously stated:

“This study aimed to assess the factors that are significant and predictive of behavior problems in dogs using the Animal Welfare Assessment Grid (AWAG) to further understand what factors influence their welfare”.

“Wilcoxon-rank sum tests were used to assess the difference in scores between dogs with behavior disorders and a cohort of healthy dogs”.

The corrected abstract appears below:

**Introduction:** Chronic pain can profoundly affect the wellbeing of dogs and our understanding is limited regarding the multidimensional impact it has on dog quality of life. This study aimed to assess the factors that are significant and predictive of chronic pain in dogs using the Animal Welfare Assessment Grid (AWAG) to further understand what factors influence their welfare.

**Methods:** Seventy six AWAG assessments were undertaken across 46 dogs that clinicians diagnosed as having musculoskeletal conditions that caused chronic pain. Wilcoxon-rank sum tests were used to assess the difference in scores between dogs with chronic pain and a cohort of healthy dogs (*n* = 143).

**Results:** All physical factors besides body condition, and all psychological, environmental, and procedural factors were significantly different between healthy dogs and dogs with chronic pain, evidencing how chronic pain impacts all domains of a dog's life. Spearman Rank Correlation Coefficient (RS) revealed several significant strong positive correlations such as the association between the severity of clinical symptoms with poorer mobility and the frequency at which the dog experienced fearful stimuli. Logistic regression showed that fears and anxieties frequency, the dog's reaction to stressors, engagement with enrichment, and social interactions were significant predictors of chronic pain in dogs.

**Discussion:** This highlights that typical signs of musculoskeletal disorders such as gait changes, stiffness, lameness might manifest after behavioral changes such as increased fearfulness, prolonged recovery from a stressful event, a reduced interested in social interactions, toys or play. Owners only seeking veterinary attention when the presence of physical signs of disease are evident may result in a delayed veterinary attention resulting in reduced welfare. Regular veterinary assessments combined with use of the AWAG can proactively identify these behavioral indicators and result in prompt treatment and improved quality of life.

The authors apologize for this error and state that this does not change the scientific conclusions of the article in any way. The original article has been updated.

