# Corrigendum: How does chronic pain impact the lives of dogs: an investigation of factors that are associated with pain using the Animal Welfare Assessment Grid

**DOI:** 10.3389/fvets.2024.1458084

**Published:** 2024-07-19

**Authors:** Rachel Malkani, Sharmini Paramasivam, Sarah Wolfensohn

**Affiliations:** School of Veterinary Medicine, University of Surrey, Guildford, United Kingdom

**Keywords:** dog, welfare assessment, quality of life, chronic pain, veterinary medicine

In the original article, there was a mistake in Figures 2, 3 as published. The term “chronic pain” in the figures was mislabeled as “behaviour”. The corrected versions of Figures 2, 3 appear below.

**Figure 2:**
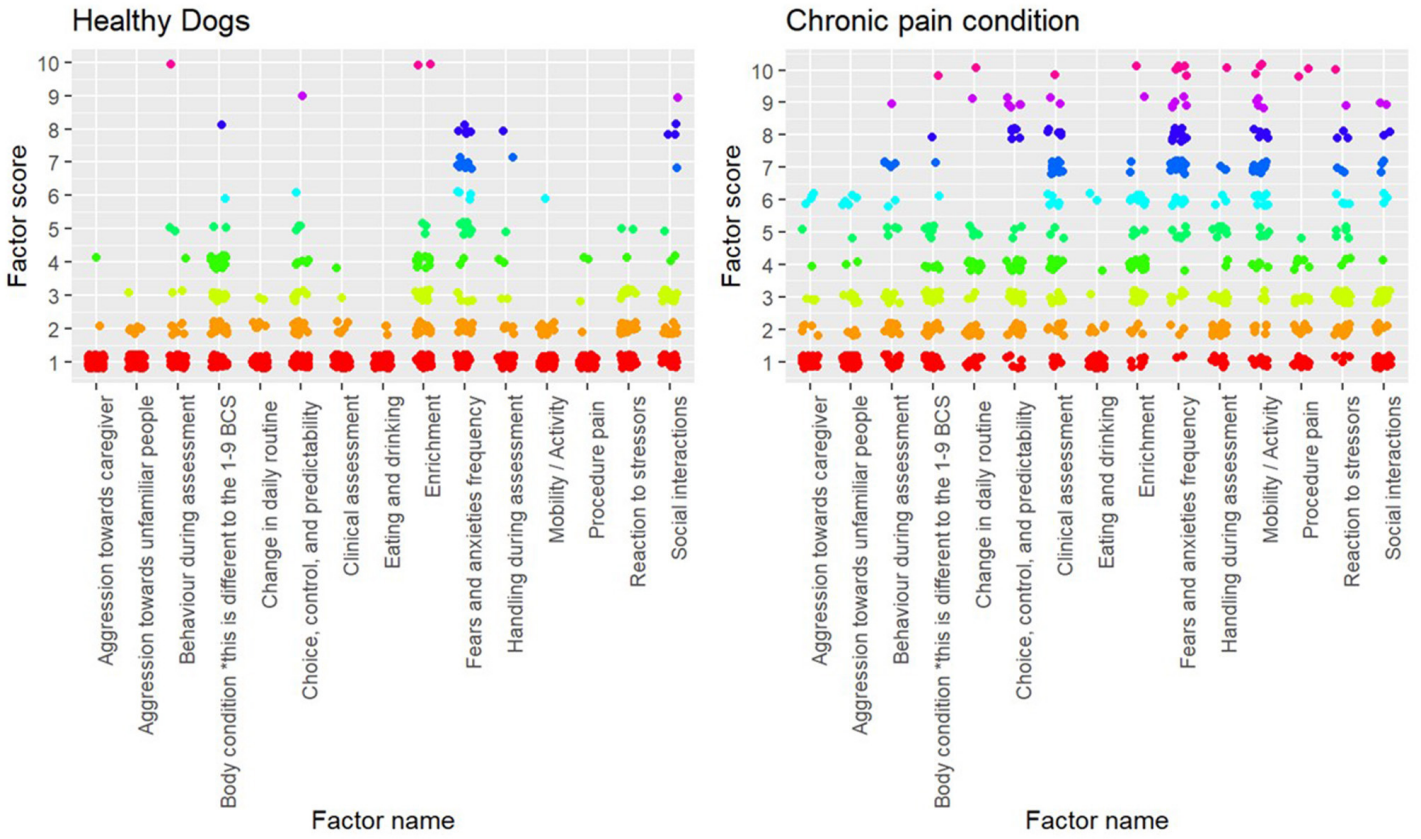
Plot of individual factor scores for each assessment (healthy, *n* = 143; chronic pain, *n* = 76).

**Figure 3:**
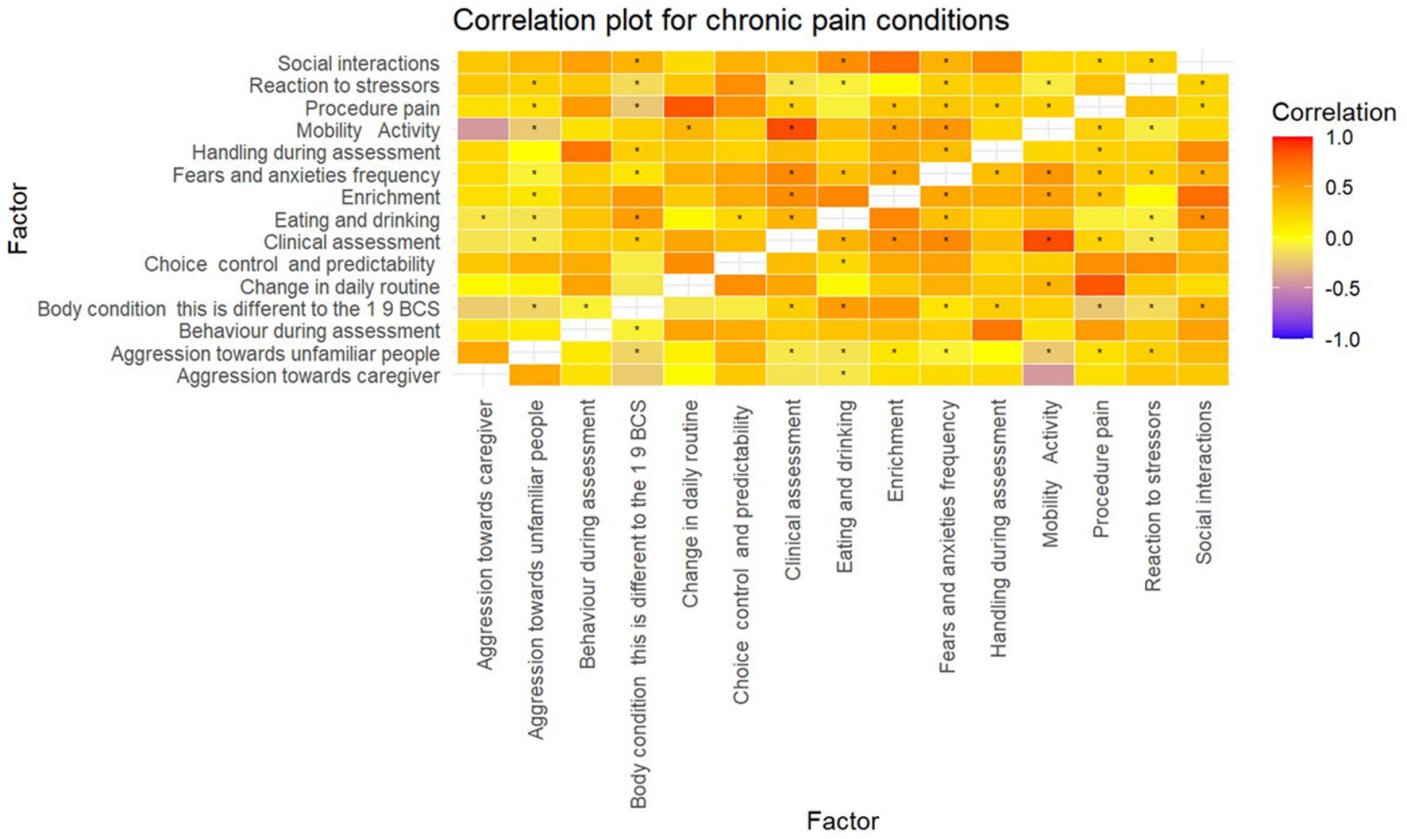
Correlation matrix for conditions that cause chronic pain. ^*^p-value < 0.05.

The authors apologize for this error and state that this does not change the scientific conclusions of the article in any way. The original article has been updated.

